# Tree Growth Under Climate Change: Evidence From Xylogenesis Timings and Kinetics

**DOI:** 10.3389/fpls.2020.00090

**Published:** 2020-02-18

**Authors:** Joana Vieira, Ana Carvalho, Filipe Campelo

**Affiliations:** Centre for Functional Ecology, Department of Life Sciences, University of Coimbra, Coimbra, Portugal

**Keywords:** cambial activity, irrigation, manipulation experiment, tracheidograms, water exclusion, wood formation

## Abstract

Tree growth is one of the most studied aspects of tree biology, particularly secondary growth. In the Mediterranean region, cambial activity is mostly determined by water availability. Climatic projections for the Mediterranean region predict more frequent and intense droughts, and longer periods without precipitation. To investigate tree growth under the predicted scenarios of climate change, a water manipulation experiment was conducted in a maritime pine stand (*Pinus pinaster* Aiton). In 2017, fifteen trees were divided into three groups: control, rain exclusion, and irrigation. Drought conditions were simulated by installing a continuous plastic sheet on the forest floor from March to September. Trees under irrigation treatment were watered twice a week in September. Cambial activity and xylem formation was monitored every 10 days from February 2017 until March 2018. Cell production was maximal around the spring equinox in all treatments. Trees under rain exclusion decreased cell production rates, xylogenesis duration, and latewood cell wall thickness. The extra irrigation in September did not produce noticeable differences in xylogenesis compared to trees in the control treatment. The synchronization of maximum cambial division rates around the vernal equinox (spring) could allow Mediterranean trees to mitigate the impact of summer drought. With the predicted increase in drought intensity and frequency, lower tree productivity, carbon sequestration, and wood biomass are expected.

## Introduction

Tree growth is one of the most studied aspects of tree biology. Through photosynthesis trees assimilate carbon, which is later stored in wood by xylogenesis (Cuny et al., 2015). Forest ecosystems play a key role in the terrestrial carbon cycle (Litton et al., 2007; Bader et al., 2013). Indeed, wood represents the principal carbon pool of terrestrial biomass (Reichstein et al., 2019), accumulated in trees through wood formation. It is noteworthy that the largest part of global vegetation biomass depends on a thin layer of cells: the vascular cambium (De Micco et al., 2019). In temperate environments, the vascular cambium presents a seasonal activity characterized by a period of activity in spring and a period of dormancy in winter (Begum et al., 2013). Besides seasonality constrains, mainly due to climate and photoperiod, cambial activity is also dependent on the rate and duration of cell production (Lupi et al., 2010; Rossi et al., 2012; Cuny et al., 2015).

The relation between the rate and duration of cambial cell production has been investigated in several studies (Lupi et al., 2010; Cuny et al., 2012; Rossi et al., 2014), with the aim of determining whether there is a dependency relation between them. Lupi et al. (2010) found that an earlier onset of xylogenesis resulted in a longer duration and higher cell production in *Picea mariana* (Miller) B.S.P. growing in Quebec. In a broader analysis, along a thermal gradient, Rossi et al. (2014) reported that cell production, rather than xylogenesis duration, explained most of the variability in growth observed in *P. mariana*. In a multi-species study, Cuny et al. (2012) estimated that 75% of the annual radial increment variability was attributed to cell production rates, and only 25% to duration. Studies in drought-prone environments have also concluded that wood production is determined by growth rates rather than by growing season length (Vieira et al., 2018; Ren et al., 2019), corroborating that wood production is mostly determined by cell division rates.

Species adjust their phenology to climate change by shifting or compressing growth and reproduction seasons according to specific regional environmental drivers, local adaptations, and individual plasticity to climate (Nord and Lynch, 2009; Diez et al., 2012). The Mediterranean climate is characterized by a summer drought that forces the cambium into a quiescent or dormant state (Cherubini et al., 2003; Vieira et al., 2014a; Vieira et al., 2015; Battipaglia et al., 2016). Xylogenesis studies in the region have observed that the growing season can stop after the summer, but, under specific climatic conditions associated with water availability, cambial activity can resume after the summer (Camarero et al., 2010; Vieira et al., 2015). The reactivation of cambial activity following the summer drought and the existence or not of a second period of cambial activity is defined as the facultative bimodal growth pattern (Campelo et al., 2018). The capacity of trees to adjust growth to the prevailing climatic conditions is gaining importance in drought prone areas where summers are becoming increasingly longer and drier.

Climate change is already a reality in the Mediterranean region, affecting the forests therein. For instance, in Portugal, the rainfall regime for the 1941–2007 period revealed that spring is getting drier and autumn wetter (Espírito Santo et al., 2014). The changes in the precipitation regime already at place in the Mediterranean region have a strong effect on tree growth. A drier spring triggers a shorter cambial activity period, by anticipating summer dormancy, and, thus, reducing spring wood production (Vieira et al., 2014a). If precipitation returns after the summer, cambial activity can resume, potentially making up for what was missed in spring. Campelo et al. (2007) showed that under specific climatic conditions, *Quercus ilex* L. can form half of the tree ring after the summer stop. Understanding climatic forcing on cambial activity is particularly important on species with two periods of growth, in order to predict how much wood is produced after the summer and to investigate whether the second growth period could represent a compensatory mechanism (Campelo et al., 2018).

Manipulation experiments allow us to reproduce the predicted scenarios of climate change and investigate trees' responses under such conditions. Climatic projections from the EURO-CODEX initiative using the Representative Concentration Pathways 4.5 (Jacob et al., 2014), predict an increase in the mean annual temperature from 3.3 to 4.1°C and a decrease of 11 to 17% of the annual total precipitation for the Mediterranean region. Precipitation is predicted to decrease in the summer months as well as the consecutive days without rain, dry days, with predictions of up to 30 more dry days per year by the end of the century (Neelin et al., 2017; Polade et al., 2014).

Maritime pine (*Pinus pinaster* Aiton) is a typical Mediterranean conifer and the third most representative forest species in Portugal. It is an economically important species (e.g., pulp, wood, resin), representing about 2% of the Portuguese National Gross Domestic Product. In Portugal, the response of maritime pine to increased spring drought has already been studied under field (Vieira et al., 2017) and greenhouse conditions (Garcia-Forner et al., 2019; Vieira et al., 2019). The rain exclusion experiment carried out in the field revealed that the rate of cell production and tracheid differentiation was reduced by the simulated drought (Vieira et al., 2017). Here, we simulate a more intense drought during the growing season and a wet autumn, in order to predict forest productivity under the expected scenarios of climate change. The hypotheses tested are i) trees show a bimodal growth pattern; ii) trees in rain exclusion present lower rates of cell production and differentiation; and, iii) trees receiving extra irrigation form latewood tracheids with wider lumen diameter.

## Materials and Methods

### Study Site and Experimental Design

A water manipulation experiment was carried out in Perímetro Florestal Dunas de Cantanhede (40°21ʹ26ʹʹN, 8°49ʹ14ʹʹW), on adult maritime pines trees (*P. pinaster* Aiton), growing on sand dunes. Climate is typically Mediterranean with oceanic influence, the average annual temperature for the area is 16.3°C and the total annual precipitation is 915 mm (1987–2016). Precipitation occurs mostly in the autumn and winter, and summer is characterized by high temperatures and low precipitation. Meteorological data (daily maximum and minimum temperature and total precipitation) were acquired from the nearest meteorological station (Instituto Português do Mar e da Atmosfera), located in Figueira da Foz, at 24 km south from the study site (40°8ʹ18ʹʹN, 8°50ʹ5ʹʹW).

In the same forest stand, three plots of five dominant trees were selected to represent three levels of water manipulation: control, irrigation, and rain exclusion. The plots were located at approximately 40 m from each other, with the control plot standing between the irrigation and rain exclusion plots. The trees presented an average diameter at breast height (DBH) of 21.0 ± 1.6 cm (F_2,12_ = 0.49, p = 0.63), 10.4 ± 1.1 m of height (F_2,12_ = 3.61, p = 0.06), and 49.8 ± 4.5 age at breast height (F_2,12_ = 2.23, p = 0.15), with no differences between treatments. Rain exclusion was performed by installing a continuous plastic sheet on the forest floor covering at least twice the tree canopy projected area on the ground (Vieira et al., 2017). The plot selected for the rain exclusion experiment was located on a mild slope which diverted the water away from the plot. The plastic sheet was installed on February 27^th^ (DOY 59) and removed on October 1st 2017 (Day Of Year, DOY, 274). The irrigation experiment was carried out in September 2017, and consisted in providing an extra 200 L of water per tree, twice a week. The extra water supplied to the trees was transported to the field in a 1,000 L water tank. The tank was connected to a motor that pumped the water out. The volume of water given to each tree was controlled by monitoring the water level in the tank. During September, each tree in the irrigation group was watered eight times, receiving a total of 1,200 L of water.

Three PR2 soil moisture probes from Delta-T-Devices were installed in the treatment plots on February 20^th^ 2017 and scheduled to record soil moisture values every 30 min at a depth of 60 cm. Data were stored in a DL2e data logger also from Delta-T-Devices. On October 15^th^ 2017 the study area was hit by a wildfire that destroyed the soil moisture probes. The fire lost intensity upon reaching the experiment site, burning only the forest floor. The study trees were not affected.

### Sample Preparation

Sampling of the cambium and developing xylem was carried out from March 2017 (DOY 62) to March 2018 (DOY 68) by collecting microcores from the tree stem every 10 days. Microcore collection was performed using a Trephor tool (Rossi et al., 2006a) starting at 45 cm above breast height in a downward spiral pattern on the south-facing side of the stem to minimize the growth variability around the stem (Lupi et al., 2014). Microcores were collected at approximately 5 cm apart to avoid getting traumatic tissue and resin ducts from previous sampling points. After collection, the microcores were placed in FAA solution (formaldehyde-acetic-acid-ethanol solution) and processed following the protocol by Rossi et al. (2006a). After paraffin embedding, the samples were cut with a rotary microtome, stained with an Astra Blue (0.15%) and Safranin (0.04%) water solution and permanently mounted using Eukitt.

### Xylem Phenology

Histological wood sections were observed under a light microscope with polarized light to distinguish each xylem differentiation phase. In cross-section, cambial cells are characterized by thin cell walls and small radial diameter. Cells in the enlargement phase present only primary wall, which does not shine under polarized light, and a diameter of at least twice that of a cambial cell. Cells in the wall-thickening and lignification phase are birefringent under polarized light, and present a bi-colored cell wall, changing from blue to red. Tracheids are considered mature when the cell wall presents a uniform red color and no cell content is observed in the lumen. Cambial, enlarging, cell wall thickening, and mature cells were counted along three radial files in order to monitor cambial activity and wood formation. Microscopic observations were performed under 400–1000 × magnification using a Leica DM4000B microscope.

The number of cells in the cambium and in the consecutive phases of xylem differentiation was fitted with generalized additive mixed models (GAMMs) to model the dynamics of cambial activity and wood formation over the growing season and to compare the effect of the water manipulation treatment (**Supplementary Table 1**). A GAMM is a generalized linear mixed model (GLMM) which uses smoothed splines fitted to the predictor variables rather than the original values of the predictor variables, i.e., extends the linear model to allow for non-linear relationships (Wood, 2006). The degree of nonlinearity is estimated by generalized cross-validation, where linear relationships are preferred over nonlinear ones. Another strength of GAMMs is the incorporation of additional random effect terms to deal with non-independence of data. In fact, mixed models should be preferred when data has more than one source of random variability, as happens with the number of cells counted more than once on the same tree (i.e., repeated measures taken over time). For this reason, tree identity was used as a random factor. By doing so, it was possible to compare the effect of treatment (three levels) throughout the growing season on xylogenesis. Differences between groups were considered significant when the pointwise confidence intervals of the fitted curves did not overlap. Analysis were performed in the R computing environment (R Development Core Team, 2019), using cubic regression splines as smoothers, with the *mgcv* package (Wood, 2006).

### Kinetics of Xylem Differentiation

For each group, the average cell number predicted by GAMMs was used to calculate the date of entrance of individual cells into each zone of cell production and differentiation (cambial, enlargement, wall thickening, and mature) over the growing season, as described by Cuny et al. (2016). These dates were later used to compute the residence time of each cell in the cambial, enlargement, and wall thickening zones. Finally, we also computed for each cell the rate of enlargement (wall deposition) by dividing its final cell diameter (cell wall width) by the duration it spent in the enlargement (wall deposition) phase.

### Tracheid Dimensions

The last microcore collected (March 2018) was processed as previously described and photographed using a camera fixed on a microscope (Leica, model DCF295), at 50 × magnification. Three radial rows of the 2017 tree ring were selected to measure tangential tracheid radial lumen diameter and cell wall thickness. Image analysis was performed on ImageJ (http://rsbweb.nih.gov/ij/).

Tracheid features were standardized to its relative position within the tree ring so that the lumen diameter and cell wall thickness could be compared between control, irrigated, and rain exclusion trees in 2017 (Campelo et al., 2016). This method determines the relative position of all tracheids by dividing the distance between the beginning of the ring and the center of each tracheid by the total ring width (Campelo et al., 2016). The standardization was performed using the method *relPos* from the package *tracheideR* in the R computing environment (Campelo et al., 2016).

Tracheids lumen diameter and cell wall thickness were compared between treatment levels using linear mixed-effects models (LMMs). Linear mixed models are an extension of simple linear models that allow both fixed and random effects, and are particularly used when there is no independence in the data, such as arises from longitudinal studies (i.e., when repeated measures were taken on the same subject over time).

A one-way analysis of variance (ANOVA) was conducted to compare the effect of treatment (three levels: control, exclusion, and irrigation) on the number of cells produced. Normality and homogeneity of variance across groups were checked using Shapiro-Wilk and Levene's tests. When ANOVAs were significant, *post hoc* comparisons were carried out using the Tukey test.

## Results

### Environmental Conditions and Soil Water Status

**Figure 1:**
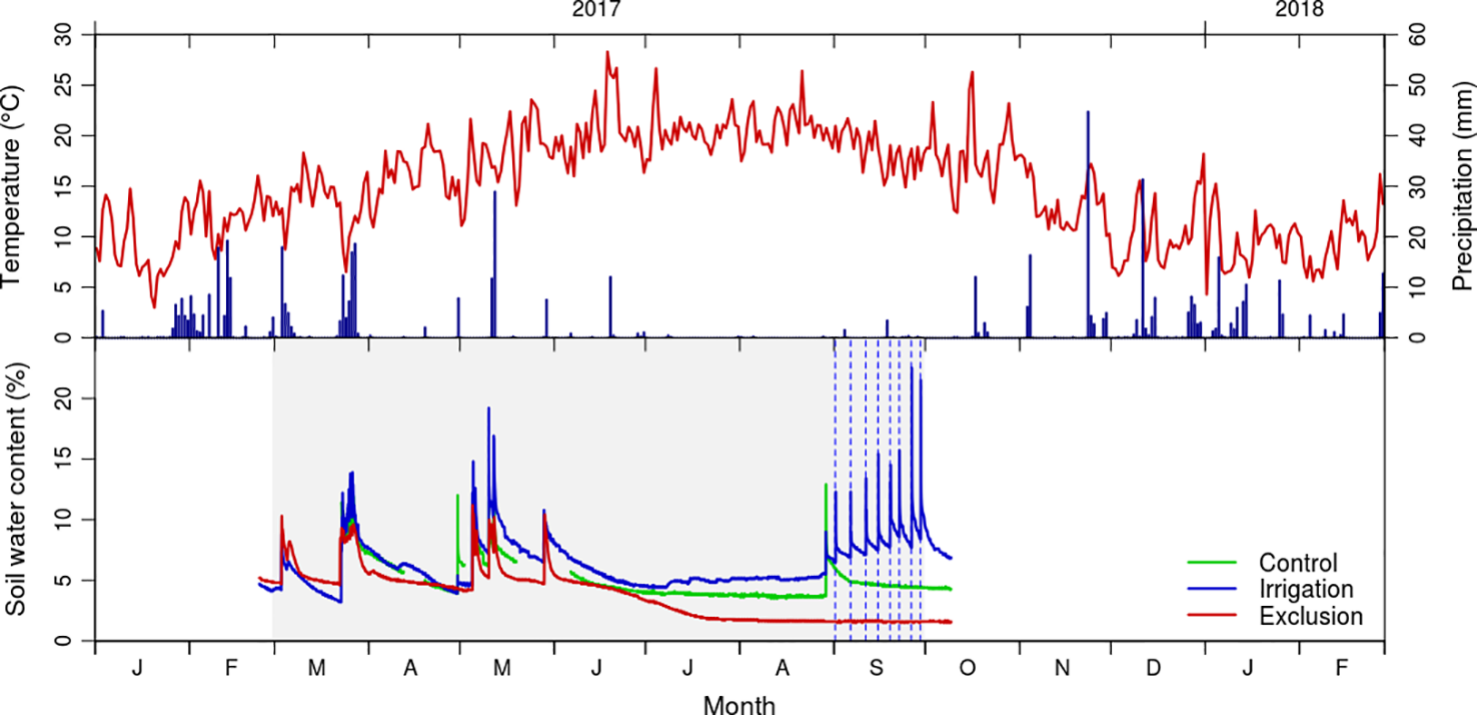
Daily values of mean temperature (°C) and total precipitation (mm) from January 2017 to March 2018 registered by the Figueira da Foz meteorological station (data from Instituto Português do Mar e da Atmosfera); and soil moisture content (volume/volume) in control (green line), exclusion (red line) and irrigation (blue line) treatment. Gray shaded area corresponds to the rain exclusion period and vertical dashed lines to the irrigation treatment.

Average annual temperature in the study area was 15.9°C in 2017, with minimum temperatures below zero (−4.4 to −0.1°C) in December 2017 and January 2018 and maximum temperatures of 38.9°C in July (**Figure 1**). Mean spring temperature was 17.4°C and mean summer temperature was 20.1°C. The study year was exceptionally dry, with a total annual precipitation of 476 mm (the long term average for the region is 915 mm). In the period prior to the rain exclusion experiment rained 123 mm, and during the rain exclusion period it rained 177 mm (**Figure 1**).

Soil water content reflected the rain events, increasing after rainfall and irrigation treatments (**Figure 1**). In the control and exclusion plots, maximum soil water content was observed in May, corresponding to 11 and 9%, whereas in the irrigation plot the soil water content reached a maximum value of 20% in September. Soil water content was minimal in the summer months, representing 6, 5, and 2% in irrigation, control, and exclusion treatments, respectively. Before the start of the irrigation treatment there was a rainfall event, with soil water content responding immediately to it in the control and irrigation plots. Soil water content progressively increased following the irrigation treatment, presenting a maximum of 20% water content after the last irrigation. At that time control presented 6% and rain exclusion 2% of soil water content. The last reading of the soil moisture probes was 10 days after removing the rain exclusion experiment, at that time soil water content was of 8, 6, and 2% in irrigation, control, and rain exclusion plots.

### Cambial and Xylem Phenology

**Figure 2:**
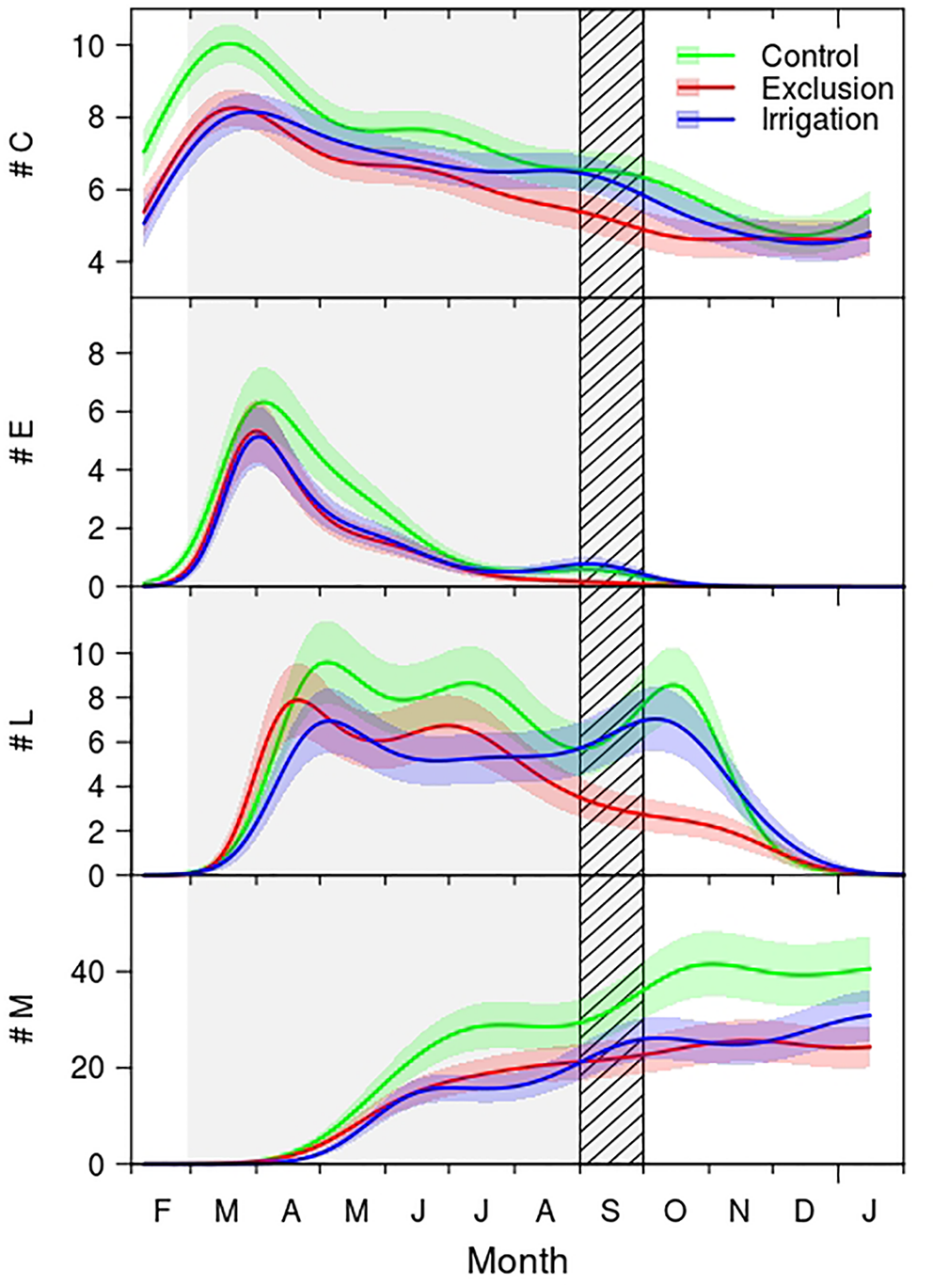
Generalized additive mixed models (GAMMs) fitted to the number of cambial (#C), enlargement (#E), cell wall thickening (#L), and mature cells (#M) of control (green line), exclusion (red line), and irrigation (blue line) trees. The solid lines are the fitted curves and the shaded colored areas the 95% confidence interval. Difference between treatments is considered significant whenever there is no overlap between the curves confidence interval areas. Gray shaded area corresponds to the rain exclusion period and diagonal lines area to the irrigation period. For detailed information on the GAMM models please see **Supplementary Table 1**.

The seasonal distribution of cambium, enlarging and mature cells presented a similar pattern between treatments (**Figure 2**), cell wall deposition, however, exhibited a clear bimodal pattern in trees under control treatment and in trees with irrigation. Trees under rain exclusion showed a unimodal pattern. The number of cells in the cambium increased during February, achieving its maximum in March. Trees in the control group presented a maximum of 10 cambial cells whereas rain exclusion and irrigation trees had on average 8 cells. From March to May the number of cells in the cambium decreased, and from there until September the number of cambial cells remained constant and similar between control and irrigation treatment trees (approximately seven cells), while the cambium of trees in rain exclusion treatment had significantly fewer cells (approximately five). From September to December the number of cambial cells decreased, reaching a minimum of five cells.

Regarding the seasonal variation of enlarging cells, the first cells in the enlargement phase were observed in mid-February, and the maximum was achieved by the end of March (**Figure 2**). At this time there were approximately six cells in trees under control treatment and five cells in trees in irrigation and in rain exclusion treatment. Control trees exhibited significantly more enlargement cells from mid-April until mid-May. The number of enlarging cells decreased until July in all groups of trees. In September there was a slight increase in the number of enlarging cells in trees in control and irrigation treatment, but not in trees under rain exclusion. Cell enlargement ended in October.

Cell wall deposition and lignification phase started in March, with trees under rain exclusion anticipating this differentiation phase by 10 days (**Figure 2**). The maximum number of cells in the cell wall deposition phase was observed in mid-April in trees with rain exclusion and in the beginning of May for control and irrigation trees. The number of cells in cell wall deposition decreased but remained constant until August, when it decreased significantly in trees under rain exclusion. Control and irrigation trees showed a second period of cell increment in October. Cell wall deposition and lignification phase ended in the end of December.

The first mature tracheids were observed in mid-April in all trees (**Figure 2**). In the end of May control trees exhibited significantly more tracheids than the trees in the other treatments, this tendency hold until the end of the growing season. In the end of the growing season trees in the control treatment formed 41 tracheids, trees under irrigation treatment 30 and rain exclusion trees 26.

### Xylogenesis Kinetics

**Figure 3:**
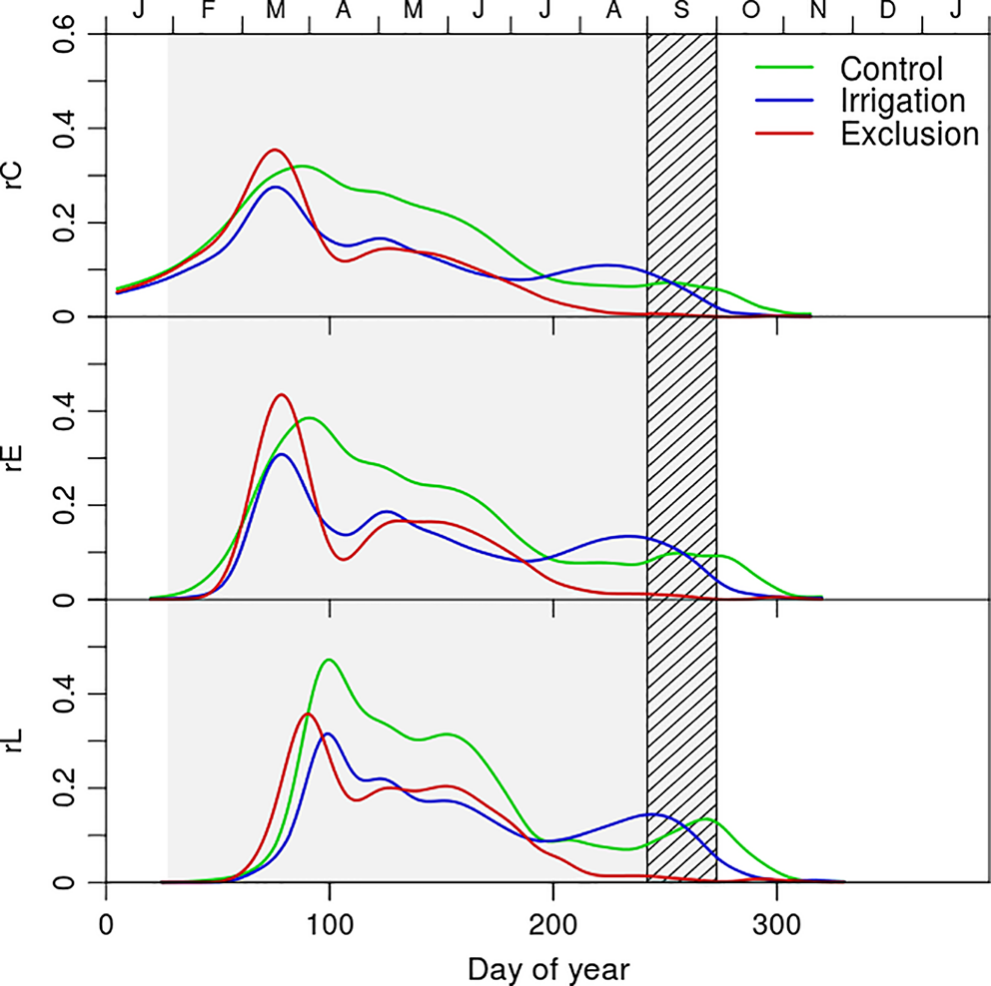
Rate of cambial cell division (rC) and rate of cell entrance in enlargement (rE), and cell wall deposition phase (rL) of control (green line), exclusion (red line), and irrigation (blue line) trees. Gray shaded area corresponds to the rain exclusion period and the diagonal line area to the irrigation period.

The rate of cell production revealed a seasonal pattern and differences between water manipulation treatments (**Figure 3**). Control trees presented a higher rate of cell production during a longer period than the remaining trees (**Figure 3**). The dynamics of cell production presented a maximum in mid-March when 0.35 tracheids were formed per day in trees under rain exclusion, 0.3 in control, and 0.28 in irrigation trees. In April trees under rain exclusion and irrigation treatments decreased their cell production rates whereas control trees maintained an increased rate of cell production until July (0.30, 0.16, and 0.15 tracheids per day in control, irrigation trees, and control trees respectively). In August, the production of new xylem cells stoped in trees under rain exclusion. At the same time, irrigation trees presented a slight increase in the rate of cell production, surpassing that of trees in control treatment (0.12 and 0.10 tracheids per day in irrigation and control trees, respectively). The entrance rate of cells in enlargement presented seasonal dynamics similar to the ones described for cambial division. The rate of cell entrance in cell wall deposition was maximal in the beginning of April for trees in irrigation and in rain exclusion treatments (0.35 and 0.30 tracheids per day in exclusion and irrigation trees, respectively). For control trees however, the rate of entrance in cell wall deposition was superior and observed in mid-April (0.48 tracheids per day). In May the first tracheids completed differentiation in all treatments. The maximum rate of tracheid maturation was higher in control (0.36, 0.30, and 0.27 tracheids per day in control, exclusion, and irrigation treatment trees, respectively). The rate of tracheid maturation decreased in September (0.09, 0.08, and 0.07 tracheids per day in irrigation, control, and exclusion treatment trees, respectively) and remained low in exclusion trees until the completion of cell deposition and tracheid differentiation in January. In trees from control and irrigation treatment however, there was an increase in the rate of tracheid maturation in November (0.2 and 0.1 tracheids per day in control and irrigation treatment trees, respectively).

**Figure 4:**
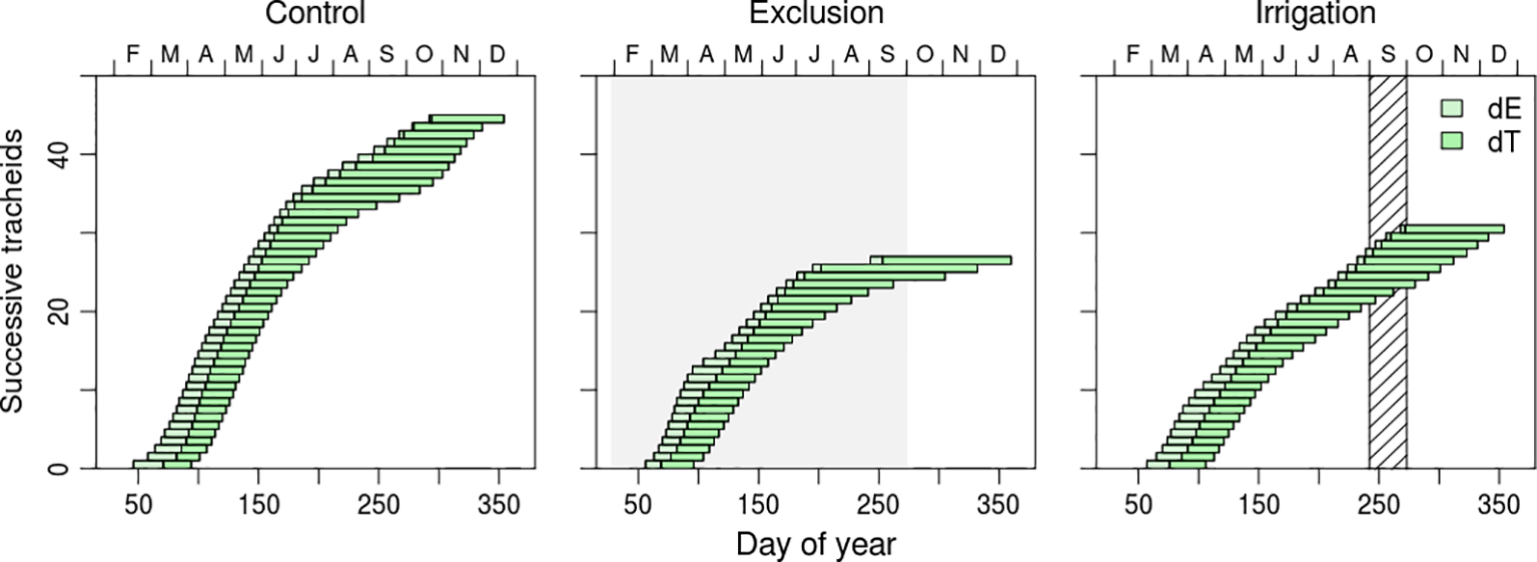
Residence time of each tracheid in enlargement (dE; light green horizontal bars) and cell wall deposition (dT; dark green horizontal bars), during the growing season of 2017. Each bar represents a tracheid. Gray shaded area corresponds to the rain exclusion period and diagonal line area to the irrigation period.

Cell residence duration in enlargement and cell wall deposition presented a seasonal variation and differences between water manipulation treatments (**Figure 4** and **Supplementary Figure 1**). Enlargement duration decreased from the first (earlywood) to the last differentiated tracheids (latewood) while cell wall thickness duration presented the opposite tendency (**Figure 4** and **Supplementary Figure 1**). Enlargement residence time of the first differentiated tracheids of control trees was of approximately 20 days whereas the last differentiated tracheids remained in enlargement for approximately 8 days. Trees in irrigation treatment presented a similar variation but trees under rain exclusion, however, presented higher residence times in enlargement, approximately 25 days. Cell wall deposition duration increased as the tracheids transitioned from earlywood to latewood (**Figure 4** and **Supplementary Figure 1**). Maximum duration of cell wall deposition was observed in the tracheids that started wall formation in July and August. The residence time of tracheids in cell wall deposition for that period was of 60, 80, and 120 days in irrigation, control, and rain exclusion treatment trees, respectively. Tracheid differentiation ended at the same time in all water manipulation treatments, at the end of December, but with different cell productions.

### Tracheid Production and Anatomy

There was a significant difference in the mean total number of cells produced (F_2,12_ = 6.53, p = 0.012) between the three treatments (**Supplementary Figure 2**). There was a significant difference between control and rain exclusion trees (p = 0.012) with trees under rain exclusion producing on average 13.1 cells less than those in the control treatments.

A significant difference in the mean number of earlywood cells produced was found between treatments (F_2,12_ = 7.61, p = 0.007); the Tukey test revealed a significant difference between trees in control and exclusion treatments (p = 0.023), with trees under rain exclusion producing on average 8.6 cells less than those in control treatment (**Supplementary Figure 2**). There was also a significant difference between trees in control and irrigation treatments (p = 0.009), with irrigation trees producing on average 10 cells less than those on the control treatment. No significant differences were observed for the number of latewood cells between treatments (F_2,12_ = 2.84, p = 0.098).

**Figure 5:**
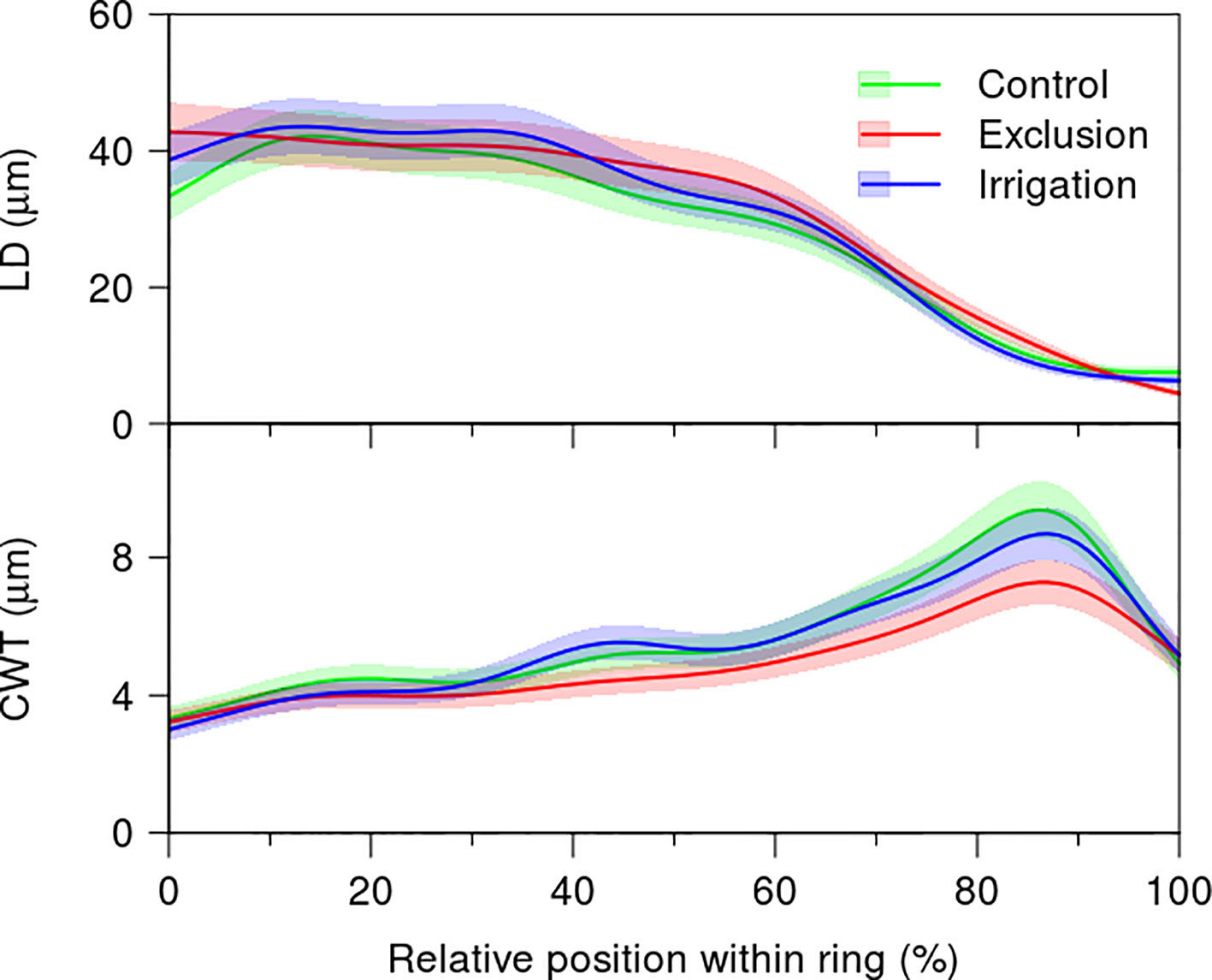
Generalized additive mixed models (GAMMs) fitted to the radial lumen diameter (LD) and cell wall thickness (CWT) tracheidograms standardized to the relative position of the tracheids within the tree ring in control (green line), irrigation (blue line), and exclusion trees (red line). For detailed information on the GAMM models please see **Supplementary Table 2**.

The variation in lumen diameter across the tree-ring did not present differences between treatments (**Figure 5**, **Supplementary Table 2**). The first 40% of the tree-ring presented a mean lumen diameter of 40 µm decreasing afterwards to a minimum of 10 µm in the last 10% of the tree-rings. Cell wall thickness increased throughout the tree-ring presenting a maximum at 90% of the tree-ring. At this point, cell wall thickness was thinner in trees under rain exclusion treatment, with a mean thickness of 6 µm, whereas trees in control and irrigation treatments presented a mean cell wall thickness of 8 µm. Cell wall thickness was significantly lower in trees under rain exclusion from 68 to 92% of the tree-ring (**Figure 5**).

**Figure 6:**
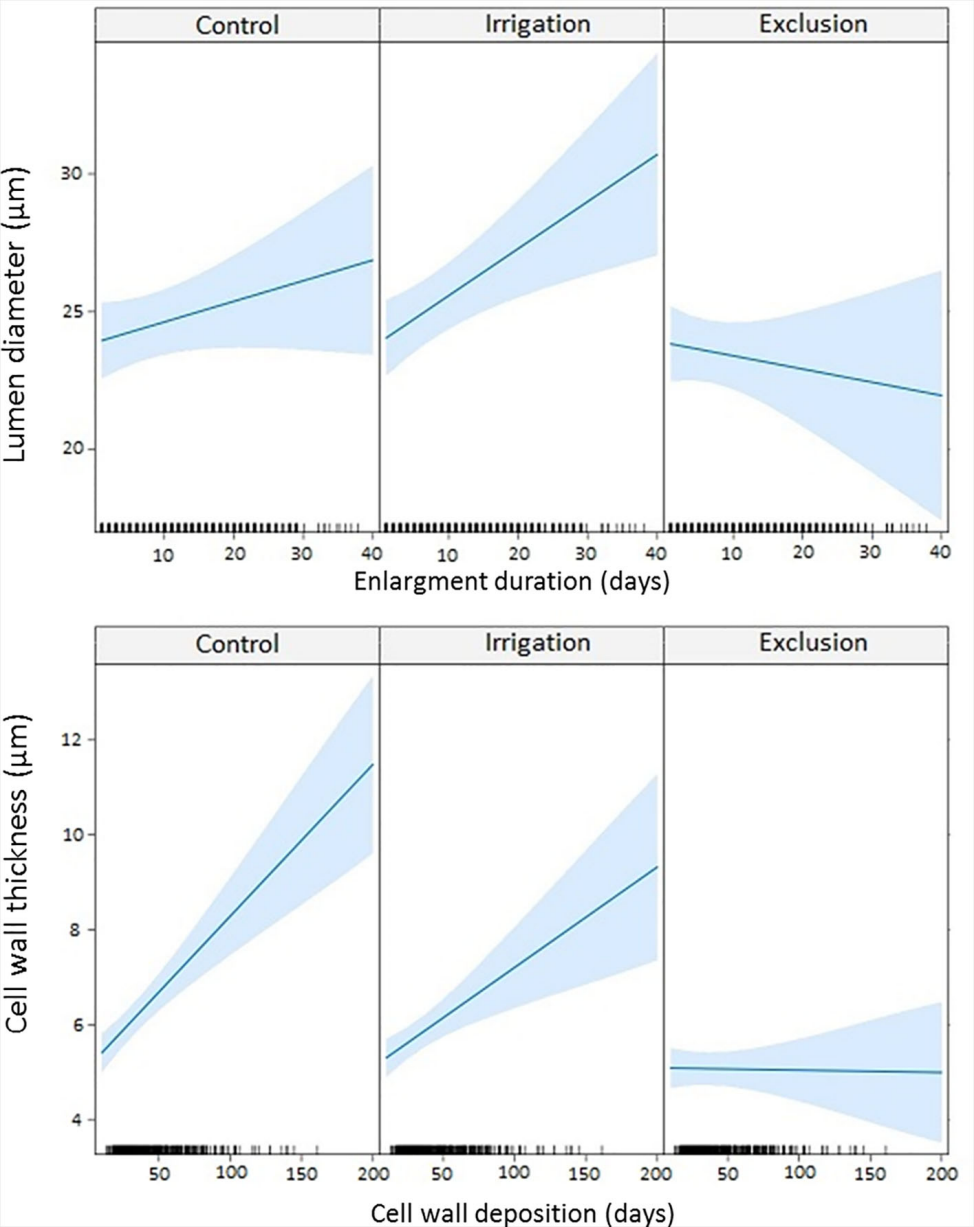
Linear mixed model (LMM) between lumen diameter and enlargement duration; and between cell wall thickness and cell wall deposition duration. Ticks in the x-axis represent the observed values.

The linear mixed-effects model (LMM) modeled lumen diameter in response to enlargement duration and cell wall thickness in response to cell wall deposition duration (**Figure 6**). A longer duration of enlargement resulted in wider lumen diameter in control and irrigation trees. In trees under rain exclusion, longer durations of enlargement did not result in wider lumen diameter. Cell wall thickness also increased with longer duration of cell wall deposition in trees in control and irrigation treatments. Rain exclusion trees presented little difference in cell wall thickness within the tree-ring. Cell wall thickness did not respond to the duration of the cell wall deposition phase.

## Discussion

This study assessed the response of cambial activity and wood formation in maritime pine trees submitted to a water manipulation experiment designed to simulate an extreme drought during the growing season and an increase in post-summer precipitation, according to the predicted scenarios for the Mediterranean region. Trees under water exclusion treatment reduced cell division rates, resulting in fewer tracheids produced with narrower cell walls. The extra irrigation in September produced no clear differences in xylogenesis between control and irrigation treatments.

### Pre-Experimental Conditions

When selecting the experimental plots, diameter at breast height (DBH) and tree height were measured to ensure that all trees were similar in size and dominance status. Although DBH and tree height did not differ between treatments, an *a posteriori* analysis of the annual growth increments revealed that irrigation trees presented significantly lower wood production in the last 20 years (**Supplementary Figure 3**). In fact, the divergence in annual wood increment started in the 1990s, when the area was strike by consecutive drought years (Barros et al., 1995). A previous study in this area revealed that after the severe drought of 1995 some trees showed a reduction in growth rates (Vieira et al., 2014b). A possible explanation for this divergence could be due to genetics, morphology (e.g., root depth), physiology, or microclimatic differences. Trees living in the same site and under identical climatic conditions may exhibit different timings and growth dynamics, leading to differences in wood production (Lupi et al., 2010; Rathgeber et al., 2011; Vieira et al., 2014b). Annual tree growth rates were not considered when selecting the trees for this experiment which could partially explain the differences between trees in control and in irrigation treatments.

### Timings and Kinetics of Xylem Formation

Maximum cell production was observed at the same time in all treatments, demonstrating that, despite the water manipulation treatment, trees were synchronized. Maximum cell production was observed at the end of March, around the vernal equinox (spring). Studies in boreal forests have shown that maximum tree growth at such latitudes is synchronized with summer solstice (Rossi et al., 2006b; Duchesne et al., 2012), a mechanism that ensures that the last differentiating tracheids have time to complete cell wall differentiation before the start of winter. A similar mechanism can be at place in the Mediterranean region, but in this case, to ensure that cell enlargement ends before the summer drought onset. Further investigation is still necessary to validate this hypothesis.

The seasonal dynamics of cambial activity and xylem differentiation presented significant differences between treatments, indicating the effectiveness of the water manipulation experiment. The rate of cell division and cell entrance in differentiation was higher in trees in control than in rain exclusion or irrigation treatments. We attribute the decreased rate of cell production of trees under rain exclusion to the reduction of soil water content. However, the low productivity of trees in the irrigation treatment must be related to the reduced competitive ability of those trees in combination with an abnormally dry year (only 75 mm of precipitation from April to August).

Besides presenting a decreased rate of cell production, cell division stopped 2 months earlier in trees under rain exclusion. Earlier stops of cambial activity in response to drought have been reported for several species growing in diverse environments (Eilmann et al., 2011; Ren et al., 2019). The adjustment of xylogenesis to environmental conditions can be achieved by adjusting the timings or the rates of cell production (Lupi et al., 2010). Earlier starts of cambial activity have been reported in response to warmer temperatures (Lenz et al., 2013; Vieira et al., 2014a) or to water availability in hyper-arid regions (Ziaco et al., 2018; Ren et al., 2019). However, most studies have reported that growth rates rather than timings determine xylem production (Balducci et al., 2016; Ren et al., 2019). The rain exclusion treatment in an extremely drought year demonstrated that, under such conditions, both rates and timings are negatively affected, significantly decreasing wood production.

### Cambial and Xylem Phenology

Water availability is the main limiting growth factor in Mediterranean forests, as reported in several dendrochronological studies (Marqués et al., 2016; Campelo et al., 2018; Gazol et al., 2018). Water limits tree growth directly by restricting the enlargement phase of tracheids during xylem formation (Cabon et al., 2019) and indirectly by reducing photosynthesis and the soluble sugars available for secondary growth (Deslauriers et al., 2017; Cartenì et al., 2018). During cell enlargement, the vacuoles of enlarging cells are loaded with sugars, increasing the turgor pressure, which attracts water toward them. It is the pressure that water exerts on the primary wall that is ultimately responsible for the expansion of the tracheid until its final size is reached (Hsiao, 1973). When modeling cell production as a function of water and carbon availability in *P. mariana* saplings, Deslauriers et al. (2016) demonstrated that water availability was ranked as the most important factor explaining total xylem cell production, while the contribution of carbon was less important.

The irrigation treatment had no noticeable effect on xylogenesis, rejecting the initial hypothesis of a second period of intense cambial activity. The resumption of cambial activity was previously associated with high precipitation in early autumn (Vieira et al., 2015; Vieira et al., 2017), causing an increase in lumen diameter of latewood cells (Carvalho et al., 2015). Our results suggest that the amount of water supplied during the extra irrigation was not enough to replicate such conditions and trigger a second period of intense cambial activity. In one of the previously mentioned studies (Vieira et al., 2015), wood formation was monitored over two consecutive years, but cambial reactivation was only observed in one of the years. In the year without cambial resumption, the amount of precipitation from June to September was very low (50 mm), as observed in our study. Our findings suggest that other factors must be involved in post-summer cambial reactivation and that the bimodal growth pattern is rather facultative than mandatory (Campelo et al., 2018). Post-summer cambial reactivation is a complex process that needs further investigation, namely to determine the role of spring—summer precipitation.

There was no increase in cell division rates following the irrigation treatment, although the rate of cell production and xylem differentiation had increased in July in trees from the irrigation treatment. The increased rate of cell production observed in July corresponded to the stop of primary growth. When primary growth stops the new shots become sources of assimilates rather than sinks, increasing the carbohydrates available for secondary growth (Deslauriers et al., 2016). An increase of cell production following the stop of primary growth was also observed in maritime pine saplings (Vieira et al., 2019), corresponding to changes in allocation from primary to secondary growth (Garcia-Forner et al., 2019).

### Tracheid Production and Anatomy

Tracheid anatomical characteristics also responded to the water manipulation experiment. Cell wall thickness was significantly thinner in trees under rain exclusion than in control and irrigation trees. Thinner cell walls result from lower carbon availability for cell wall formation (Cartenì et al., 2018). Trees growing under environmental constraints such as drought, can present growth restrictions before carbon shortages come into play (Palacio et al., 2014). Under limiting availability of assimilates, carbon storage is given priority over growth because, ultimately, tree survival depends on carbon demands for metabolism rather than for growth (Sala et al., 2012; Palacio et al., 2014). A reduction in cell wall thickness in response to drought was also reported in *Pinus nigra* Arn. and *Pinus sylvestris* L. growing in east-central Spain (Martin-Benito et al., 2017).

Similar variation in lumen diameter within the tree ring was found between treatments. However, a relationship between tracheid traits and differentiation duration was not observed. Trees in control and irrigation treatments presented a positive relationship between lumen diameter and enlargement duration, and between cell wall thickness and cell wall deposition duration, as reported by Cuny et al. (2014). In rain exclusion trees, however, there was a negative relation between lumen diameter and enlargement duration and no relation between cell wall thickness and cell wall deposition duration. The water stress was so intense that tracheids spent four times longer in enlargement to reach the same size as tracheids from trees under control or irrigation treatments (**Supplementary Figure 1**). Trees under rain exclusion overcame water limitation during enlargement by increasing its duration. Likewise, a non-linear relationship between temporal dynamics of cell differentiation and cell traits, where cell lumen and cell wall thickening remained stable after reaching a plateau, regardless from differentiation's duration was reported by Buttò et al. (2019).

## Conclusions

This study compared the timings and kinetics of xylem formation in maritime pine trees subjected to a field manipulation experiment designed to simulate the predicted scenarios of climate change for the Mediterranean region. The results showed that trees subjected to rain exclusion reduced both duration and rates of xylem cell production, resulting in a decline in wood production. The extra irrigation given in September was insufficient to trigger a second period of intense cambial activity. Although there were differences in the timings and rates of xylem cell production between treatments, cell production rates were maximal around vernal equinox (spring). This synchronization could be a safety mechanism designed to ensure that cell enlargement ends before the onset of summer drought. The predicted increase in drought frequency and intensity will have a detrimental effect on tree productivity, decreasing carbon fixation and wood biomass production. The knowledge gathered from such experiments can be used to improve forest management programs for climate change mitigation.

## Data Availability Statement

The datasets generated for this study are available on request to the corresponding author.

## Author Contributions

FC and JV designed the study and proposed the hypothesis tested. AC and JV analyzed the samples. FC analyzed the data. JV and FC wrote and revised the manuscript. All authors read and approved the final manuscript.

## Funding

This study was financed by the Fundação para a Ciência e a Tecnologia, Ministério da Educação e Ciência (FCT/MEC) through national funds and the co-funding by FEDER, within the PT2020 Partnership Agreement and COMPETE 2020, through the projects PTDC/AAG-GLO/4784/2014 and UID/BIA/04004/2019.

## Conflict of Interest

The authors declare that the research was conducted in the absence of any commercial or financial relationships that could be construed as a potential conflict of interest.
