# Supplementary material for: Tree Growth Under Climate Change: Evidence From Xylogenesis Timings and Kinetics
Source: Front Plant Sci. 2020 Feb 18;11:90. doi: 10.3389/fpls.2020.00090 (PMC7040628; doi:10.3389/fpls.2020.00090)
Supplement: Supplementary file 1 [file DataSheet_1.docx]

**Supplementary material**

**
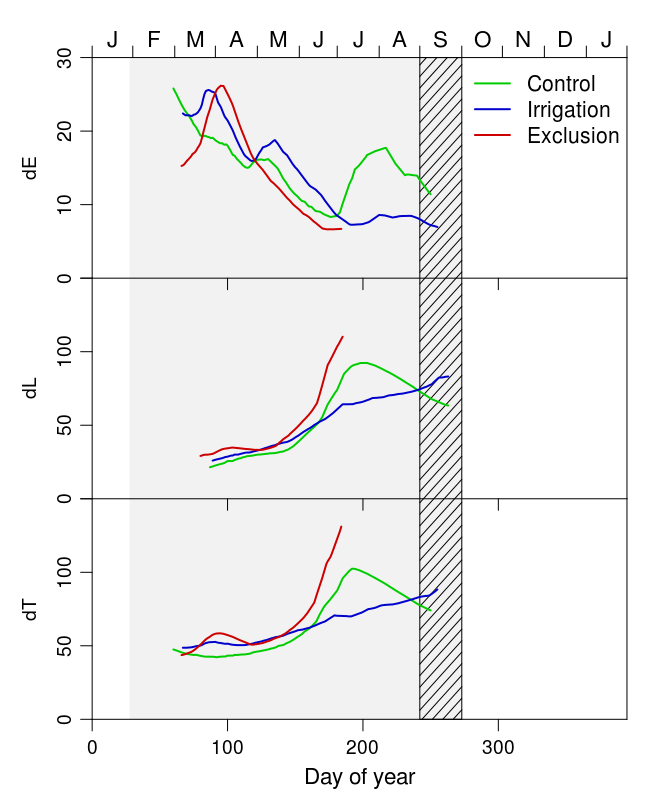
**

**Supplementary Figure 1.** Seasonal variation in the duration (in days) of enlargement phase (dE), cell wall deposition phase (dL) and total duration (dT), of control (green line), exclusion (red line) and irrigation (blue line) trees. Grey shaded area corresponds to the rain exclusion period and diagonal line area to the irrigation period.

**
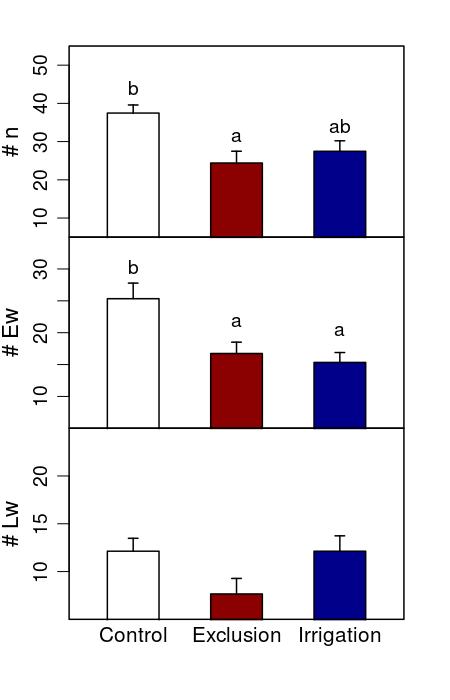
**

**Supplementary Figure 2.** Total, earlywood and latewood tracheids formed in control (white bar), exclusion (red bar) and irrigation trees (blue bar). Letters represent significant differences between treatments determined with a one-way ANOVA. Whiskers represent standard deviation.

**
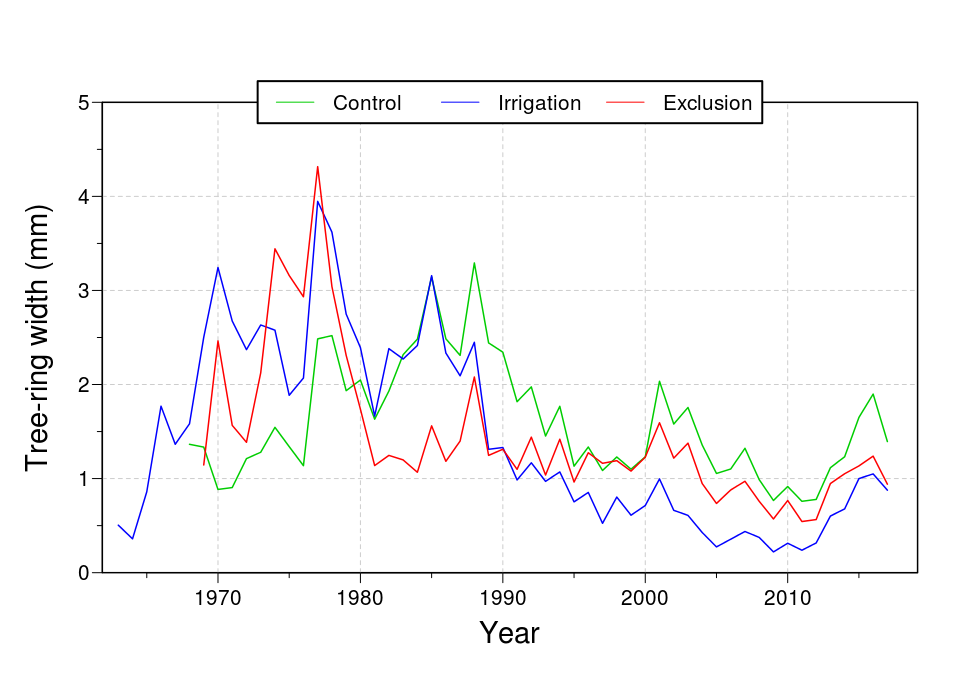
**

**Supplementary Figure 3.** Raw tree-ring width chronologies of control (green line), exclusion (red line) and irrigation (blue line) maritime pine trees (n=5). Cores were collected in May 2018 and processed following standard dendrochronology procedures. Tree-ring widths were measured using the R package xRing (Campelo et al., 2019).
